# Adherence to isoniazid prophylaxis among HIV-infected children: a randomized controlled trial comparing two dosing schedules

**DOI:** 10.1186/1741-7015-7-67

**Published:** 2009-11-03

**Authors:** Stanzi M le Roux, Mark F Cotton, Jonathan E Golub, David M le Roux, Lesley Workman, Heather J Zar

**Affiliations:** 1 School of Child and Adolescent Health, University of Cape Town, South Africa; 2 Department of Paediatrics and Child Health, Stellenbosch, South Africa; 3 Department of Epidemiology, Johns Hopkins School of Medicine & Bloomberg School of Public Health, USA

## Abstract

**Background:**

Tuberculosis contributes significantly to morbidity and mortality among HIV-infected children in sub-Saharan Africa. Isoniazid prophylaxis can reduce tuberculosis incidence in this population. However, for the treatment to be effective, adherence to the medication must be optimized. We investigated adherence to isoniazid prophylaxis administered daily, compared to three times a week, and predictors of adherence amongst HIV-infected children.

**Methods:**

We investigated adherence to study medication in a two centre, randomized trial comparing daily to three times a week dosing of isoniazid. The study was conducted at two tertiary paediatric care centres in Cape Town, South Africa. Over a 5 year period, we followed 324 HIV-infected children aged ≥ 8 weeks. Adherence information based on pill counts was available for 276 children. Percentage adherence was calculated by counting the number of pills returned. Adherence ≥ 90% was considered to be optimal. Analysis was done using summary and repeated measures, comparing adherence to the two dosing schedules. Mean percentage adherence (per child during follow-up time) was used to compare the mean of each group as well as the proportion of children achieving an adherence of ≥ 90% in each group. For repeated measures, percentage adherence (per child per visit) was dichotomized at 90%. A logistic regression model with generalized estimating equations, to account for within-individual correlation, was used to evaluate the impact of the dosing schedule. Adjustments were made for potential confounders and we assessed potential baseline and time-varying adherence determinants.

**Results:**

The overall adherence to isoniazid was excellent, with a mean adherence of 94.7% (95% confidence interval [CI] 93.5-95.9); similar mean adherence was achieved by the group taking daily medication (93.8%; 95% CI 92.1-95.6) and by the three times a week group (95.5%; 95% CI 93.8-97.2). Two-hundred and seventeen (78.6%) children achieved a mean adherence of ≥ 90%. Adherence was similar for daily and three times a week dosing schedules in univariate (odds ratio [OR] 0.88; 95% CI 0.66-1.17; *P *= 0.38) and multivariate (adjusted OR 0.85; 95% CI 0.64-1.11; *P *= 0.23) models. Children from overcrowded homes were less adherent (adjusted OR 0.71; 95% CI 0.54-0.95; *P *= 0.02). Age at study visit was predictive of adherence, with better adherence achieved in children older than 4 years (adjusted OR 1.96; 95% CI 1.16-3.32; *P *= 0.01).

**Conclusion:**

Adherence to isoniazid was excellent regardless of the dosing schedule used. Intermittent dosing of isoniazid prophylaxis can be considered as an alternative to daily dosing, without compromising adherence or efficacy.

**Trial registration:**

Clinical Trials NCT00330304

## Background

In 2007, 90% of the estimated 2.0 million HIV-infected children worldwide lived in sub-Saharan Africa, a reflection of the HIV epidemic in adults of the same region [1]. Opportunistic infections, especially tuberculosis (TB), are a major cause of morbidity and mortality amongst these children [2,3]. Internationally, significant progress has been made to minimize morbidity and mortality amongst people living with HIV/AIDS. The benefits of highly active antiretroviral therapy (HAART), trimethoprim-sulphamethoxazole (TMP-SMX) prophylaxis and, more recently, isoniazid (INH) prophylaxis for preventing TB, have been proven in adults and children in various settings [4-8]. However, in resource-limited settings these benefits are rarely realized. Obstacles include cost, interrupted drug supplies and suboptimal adherence [1,9,10].

To enable all those infected by HIV to benefit from these life-saving interventions, it is imperative that cost-effectiveness and barriers to adherence be urgently and innovatively addressed. Common barriers to adherence include complex dosing schedules, toxicity, pill burden and, in many resource-limited settings, financial cost to the patient [11,12]. Intermittent dosing schedules can potentially address these adherence barriers and may prove cost-saving. Intermittent dosing for the treatment and prevention of TB has been successfully used for adults and efficacy has been proven in children [8,13-15]. Even if the efficacy of a treatment is similar for different dosing schedules, it is possible that subtle adherence differences may impact on the feasibility and effectiveness when the treatment is rolled out on a large scale. To our knowledge, there have been no randomized controlled trials evaluating the impact of dosing schedule on adherence to INH prophylaxis in HIV-infected children.

In a recent placebo-controlled trial evaluating INH prophylaxis for HIV-infected children in Cape Town, South Africa, INH markedly reduced mortality and TB incidence with no difference in the efficacy between a daily and an intermittent dosing schedule [8]. Using the same study cohort, we describe the overall adherence rates as measured by pill counts and caregiver self-reports. We investigate the potential differences in adherence between the two dosing schedules and assess the predictors of adherence.

## Methods

### Design

We assessed the adherence to study medication in a randomized, placebo-controlled trial evaluating the impact of INH therapy on TB incidence and mortality among HIV-infected children. The trial had a factorial design with two levels of randomization: participants were randomized to either INH or placebo, which were given either daily or three times a week. TMP-SMX prophylaxis was administered on the same dosing schedule as INH/placebo. Enrolment for the study began in December 2002 at two tertiary paediatric care centres in Cape Town, South Africa. Enrolment continued until March 2005. The initial funding allowed for up to 2 years of follow-up. Subject to the availability of funding, further follow-up (and the continuation of INH) was to be considered by the study data and safety monitoring board (DSMB), based on interim analyses of the main outcomes, provided that caregiver consent and further ethics committee approval was obtained. As reported previously, interim analysis of the main outcomes showed a significant early survival benefit for children receiving INH [8]. On the recommendation of the DSMB, the placebo arm was discontinued on 17 May 2004. With caregiver consent and approval by the relevant ethics committees, children who had been randomized to receive placebo were then commenced on INH, either daily or three times a week, and the follow-up time was prolonged until a further assessment by the DSMB. The trial continued to assess the potential differences in efficacy, toxicity and adherence between the groups randomized to daily or three times a week INH and TMP-SMX. The final DSMB recommendation was to discontinue INH for all patients at the earliest study visit after 31 December 2007.

### Setting and participants

The study was conducted at the Red Cross War Memorial Children's Hospital (University of Cape Town) and Tygerberg Children's Hospital (Stellenbosch University), two tertiary paediatric care centres in Cape Town, South Africa. HIV-infected children older than 8 weeks of age were recruited from inpatient- and outpatient settings at the two study sites, as well as from nearby referral hospitals and clinics. HIV status was confirmed at enrolment by polymerase chain reaction (Amplicore HIV-1, Roche Diagnostic Systems) in children aged 15 months or younger and by two enzyme linked immunosorbent assays (Abbot AxSYM HIV antibody/antigen ELISA) in older children. The inclusion and exclusion criteria have already been described [8] and included residence in the Cape Town Metropolitan area with access to public or other transport. Written informed consent was obtained from a parent or legal guardian. Randomization procedures and blinding of INH/placebo allocation have already been described [8]. Researchers collected socio-demographic, clinical and laboratory data at enrolment and at scheduled follow-up visits. Study pharmacists dispensed the study drugs at each follow-up visit. The children were seen every 4 weeks for the first 6 months, every 6 weeks for the next 6 months and then every 2-3 months, depending on medical and social circumstances. At the start of the study, HAART was not widely available to South African children, but it had been accessed for some through charitable donations or participation in pharmaceutical trials. During the study period, the South African government initiated a public sector anti-retroviral programme and all study participants who qualified on medical and social criteria (as defined by national guidelines) were commenced on HAART.

### Ethical approval and monitoring

A data and safety monitoring board, comprising international and South African experts, was established to review the safety and progress of the study based on 3 to 6-monthly interim data analyses. The study was conducted in compliance with the Declaration of Helsinki. Ethical approval was given by the research and ethics committees of the Universities of Cape Town and Stellenbosch, South Africa. This secondary analysis was also approved by the Johns Hopkins School of Medicine Institutional Review Board.

### Intervention

Children were randomized to receive INH or placebo either daily or three times a week (Monday, Wednesday and Friday). INH tablets (100 mg, Be-Tabs Pharmaceuticals, Johannesburg, South Africa) were given at a dose of 10 mg/kg with range of 8-12 mg/kg depending on whether half or quarter tablets were required. INH was not given in syrup form as this has potential long-term toxicity from additives such as chloroform, requires reconstitution by a pharmacist and, due to the higher cost, is not readily available in resource-limited settings. Placebo tablets were manufactured to look identical to INH tablets. TMP-SMX was administered according to the same dosing schedule as INH/placebo, at a dosage of 5 mg/kg of the trimethoprim component.

### Adherence monitoring and support

All caregivers were issued monthly treatment diaries as an aid to adherence. The diaries were provided in English, Afrikaans or isiXhosa, depending on caregiver preference. Caregivers were requested to tick 'yes' or 'no' for each day of the month, indicating whether or not medication was administered on that day. Multiple diaries were supplied as needed. Space was provided for notes detailing any difficulties in administering the drugs. Illiterate caregivers were shown how to tick 'yes' or 'no' for each day medication was given (Additional file 1). Caregivers were instructed to return all empty medicine containers and any medication left over at each visit, along with the completed diaries. At each visit, caregivers were given the opportunity to discuss any difficulties regarding medication administration. Consultations occurred in English, Afrikaans or isiXhosa, depending on the caregiver's preference. To accommodate unforeseen difficulties in attending visits, up to 1 week's extra supply of study medication was issued at each visit. Meals and compensation for travel costs were provided on the scheduled study visits. If a child missed a scheduled visit, attempts were made to contact their caregiver. If caregivers were not able to be contacted by telephone and/or did not return for a rescheduled visit, community workers contacted them in person. A minimum of three attempts at contacting caregivers was required over a period of 3 months before a child was declared lost to follow-up.

For the first 11 months of the study adherence was assessed by caregiver self-report only. Caregivers were asked how many doses of medication had been taken in the previous week, starting from the day before the visit. From October 2003 to February 2005, both caregiver reports and pill counts were used to measure adherence. Adherence monitoring by pill counts continued until March 2008, when the last study participant discontinued INH. At each scheduled study visit, study pharmacists calculated the number of tablets that were presumably administered to the child by subtracting the number of pills returned from those dispensed. This number was then expressed as a percentage of the number of tablets that should have been given to the child. Study team pharmacists, doctors and/or nurses counselled all caregivers on adherence at the regular study visits and provided immediate feedback on pill count calculations.

### Statistical analysis

#### Outcome measures and predictors

We used various methods to explore potential adherence differences between dosing schedules. Summary measures of adherence were used to assess overall adherence and for simple comparisons of adherence between the groups. Mean adherence per child over follow-up time was compared between groups using a two-sample *t*-test with Satterthwaite's modified degrees of freedom. The proportion of children achieving mean adherence ≥ 90% was also compared between groups. Although completing 80% of prescribed doses is the conventional standard for adherence assessment in trials of TB prophylaxis [16], both self-report measures and clinic-based pill counts have been reported to overestimate adherence [17-19]. Furthermore, adherence of 95% is considered optimal in the context of HAART [20], although a cut off of 90% is often used. Given our study population, we chose a more conservative cut-off of 90% to indicate adherence to INH. Caregiver self-report and pill count determined adherence measures were analysed separately for group effect. Caregiver reported adherence was transformed into a percentage adherence by dividing the reported number of doses given by the number of doses that should have been given in the previous week. We explored agreement between caregiver report and pill counts using binary indicators cut at 90%.

The effect of time on mean adherence was visualized by comparing mean adherence per year between four year categories: 0-12 months; 12-24 months; 24-36 months; and > 36 months on study. Mean adherence per child per year was used to calculate overall mean adherence per year.

Death and lost-to-follow up might represent non-adherence in the extreme and are not accounted for by pill counts or self-report. We therefore compared these between groups using the chi squared statistic with Fisher's exact test. We also compared the mean number of admissions and the number of children who were diagnosed with TB between the groups.

As more pill return measures than caregiver self-reports were available, and over a longer period of time, we chose percentage adherence to INH/placebo as determined by pill counts as our main outcome measure. We chose to dichotomize percentage adherence in order to use logistic regression as assumptions for linear regression did not hold. Our primary independent variable was the dosing schedule (daily versus three times per week), hereafter referred to as the prophylaxis group. In order to allow both baseline and time-varying covariates to be explored as potential determinants of adherence, we applied a binary logistic regression model with generalized estimating equations (GEE) and robust variance estimation to account for the within-patient correlation of adherence measures. An independence working correlation was assumed [21].

Characteristics assessed at enrolment included the prophylaxis group (daily or three times a week), study site, gender, availability of amenities and the number of individuals per household. We chose to model child age as dynamic, using age at pill count visit rather than age on enrolment. Other potential time-varying adherence determinants (indicating status at time of adherence measurement) were: study drug (INH or placebo); severity of disease (HIV clinical and immunological staging); time on study; and concurrent use of HAART. We categorized HIV disease severity according to the Centers for Disease Control and Prevention (CDC) clinical and immunological staging criteria; categories N and A (mild clinical disease) were combined for the analysis, as there were only a few children in category N. We fitted locally weighted scatterplot smoothing (lowess) models for each continuous predictor against the logarithm of adherence to establish the general form of the relation. The age of a child was categorized as: (1) less than 1 year; (2) 1-4 years; or (3) over 4 years. Time on study was dichotomized at 1 year. The number of individuals per household was dichotomized at five (median number per household). Collinearity was checked with variance inflation factors. Univariate logistic regression with GEE was used to assess total effects of variables on adherence. Variables that were associated with adherence at a level of *P *< 0.1 were included for multivariate analysis. Variables lacking significant association with the outcome, which have previously been identified as independent predictors of adherence in the published literature, were also retained in the model (HIV clinical and immunological disease severity; concurrent use of HAART; gender; length of time on study). The study site was also forced in the model to adjust for unmeasured confounders between sites. Potential interactions between the prophylaxis group and all other covariates were explored. The model fit was further assessed using Akaike's information criteria. The statistical unit for the longitudinal analysis is the child; odds ratios < 1 indicate worse adherence. All *P*-values are two-tailed. Data were analysed using Stata statistical software, version 10 (Stata) [22].

#### Sample size

We present the *a priori *sample size calculations based on the primary objectives of the overall study. For the primary outcomes of the two levels of the study - namely mortality comparing INH to placebo (superiority) and mortality comparing daily to three times per week TMP-SMX (equivalence), sample sizes of 216 per arm (432 children overall) - was calculated to achieve power ≥ 80%, allowing for a 10% drop out. Using a survival analysis approach, a sample size of 196 children per arm would have allowed a 0.05 level one-sided log-rank test to have 80% power to detect the difference between a placebo mortality proportion at time t of 0.100 and an INH mortality proportion at time t of 0.050 (constant hazard ratio of 0.769), assuming no drop outs before time *t*. With 200 subjects in each group, the lower limit of the observed one-sided 95% confidence interval was expected to exceed -0.1 (a difference in mortality of less than 10%) with 95% power when the standard survival proportion was 0.9 and the test expected survival proportion was 0.9.

The *a priori *study design did not include a sample size calculation for this secondary analysis. The sample size (*n *= 276) for our main analysis was necessarily limited to children whose caregivers provided medication returns from which we could calculate the pill counts. Based on previous literature, we expected adherence rates of 60%-80% to daily INH. We chose *a priori *to define an adherence difference of ≥ 10% as clinically significant. With a sample size of 276 (*n*1 = 129, *n*2 = 148), we have 100% power to detect a 10% difference between a mean percentage adherence of 70 and a mean percentage adherence of 80. (Additional file 2)

## Results

### Study population

**Figure 1:**
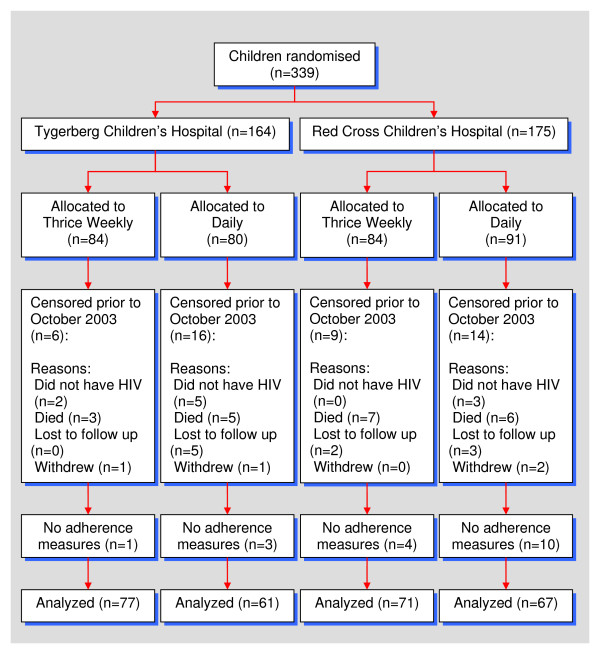
**Flow of participants through trial**.

A total of 339 children were enrolled in the study. Fifteen were excluded from the study soon after enrolment (10 tested HIV-negative on confirmatory tests and five were lost to follow-up within 1 month). For the main analysis of adherence using pill count measures, we further excluded 30 who were censored from the study before pill count based adherence monitoring started in October 2003 (Figure 1). Of the 294 children who were uncensored by October 2003, adherence percentage calculations were available for 276; the baseline characteristics of these children are shown in Table 1. About half of the children were boys. The median age at enrolment was 26 months, with 25% of the children younger than 12 months. Only three were older than 10 years when they joined the study. Most had symptomatic HIV disease, either CDC clinical category B or C. Similarly, just over 70% were classified as having moderate to severe immunological impairment (CD4 count of < 25% total lymphocyte count). Twenty-eight (10%) were on HAART at enrolment and a further 170 (61.6%) started HAART during the study. A large proportion had relatively poor socio-economic circumstances. Just under half of the children did not live in brick houses but in temporary dwellings. Although electricity was available to the majority, 52.2% did not have tap water in their house and 56% did not have ready access to a flush toilet. The median number of people sharing a house was five, with 35.6% of the children coming from households containing more than five people.

**Table 1:** Baseline comparison of children randomized to daily and three times a week dosing schedule.

**Baseline characteristics**	**Daily** **(*n *= 128)**	**Three times a week (*n *= 148)**	**Total** **(*n *= 276)**	P* Value
Sex (%)				
Male	57.0	55.4	56.3	0.81

Age (months)				0.45
Median	29.7	21.8	25.9	
(IQR)	(13.1 to 47.7)	(10.2 to 52.6)	(11.9 to 51.0)	

Age (years; %)				0.50
< 1 year	23.4	26.4	25.0	
1-4 years	51.6	44.6	47.8	
> 4 years	25.0	29.0	27.1	

Study drug (%)				0.39
Placebo	35.2	40.5	38.0	
Isoniazid	64.8	59.5	62.1	

Site (%)				0.55
RCCH	52.3	48.0	50.0	
TCH	47.7	52.0	50.0	

CDC clinical stage (%)				0.95
N or A	14.8	13.5	14.1	
B	65.6	66.9	66.3	
C	19.5	19.6	19.6	

CDC immune stage (%)				0.09
1	22.7	34.5	29.0	
2	46.1	37.2	41.3	
3	31.2	28.4	29.7	

On HAART at randomization (%)	10.9	9.5	10.1	0.7

Started HAART during study (%)	64.8	58.8	61.6	0.32

No tap water in house (%)	50.0	53.7	52.2	0.63

No electricity in house (%)	16.5	23.7	20.3	0.18

No flush toilet in house (%)	55.1	56.8	56.0	0.81

Not a brick house (%)	41.7	45.6	43.8	0.54

Number of people living in house				0.5
Med	5	5	5	
(IQR)	(3 to 6)	(4 to 6.5)	(3 to 6)	

Number of people in house (%)				0.31
≤ 5	67.7	61.5	64.4	
> 5	32.3	38.5	35.6	

Figures are medians (interquartile range) for continuous variables and percentage of children for categorical variables.

* *P*-values from Fisher's exact test for categorical data or two-sample Wilcoxon rank-sum (Mann-Whitney) test for continuous data

RCCH = Red Cross War Memorial Children's Hospital; TCH = Tygerberg Children's Hospital; CDC = Centers for Disease Control and Prevention; HAART = highly active antiretroviral therapy.

Ten children had measurements for caregiver self-reported adherence only; they were all censored before pill count measures were introduced. Their baseline characteristics were as follows: seven received daily prophylaxis; the median age was 11 months; and there were four boys. All 10 children were classified as either CDC clinical stage B or C; 8 were either CDC immune category 2 or 3. Only three received HAART before censoring. (Two more voluntarily withdrew from the study as they were due to start HAART, two were lost to follow up within three months and one child relocated. Four children died; three of these were on placebo at the time). The same number had access to water and electricity (6); the median number of individuals per household was 5. Four children lived in brick houses and the same number had access to a flush toilet. Half of the children were on placebo; equal numbers came from each study site.

### Follow-up, attrition and disease

Over 5 years from December 2002 to March 2008, the mean follow-up time was 25.3 months (standard deviation [SD] 16.3 months). The average time receiving INH prophylaxis was 22.5 months (SD 16.3 months); 75% of children received 12 or more months of INH. Of the 291 children who should have been receiving INH (that is, excluding children randomized to placebo and censored prior to unblinding), only 35 (12%) did not complete 6 months of INH: 25 had died, four were lost to follow-up and six had withdrawn from the study. Three of these children had relocated. Eighteen of the 35 had been allocated to daily prophylaxis.

Overall, 28 (8.6%) of the 324 children were lost to follow-up during the study period (mean follow-up time 18 months, similar between groups *P *= 0.81); the majority of these (75%) were followed for at least 1 year. Numbers of deaths and withdrawals were also similar in both study groups. Fifty-three children died, 29 of whom (55%) were in the daily group (*P *= 0.45). Thirty-eight were withdrawn from the study; 20 of these were in the daily group (*P *= 0.73). Two-thirds of the withdrawals were due to logistical difficulties for the caregiver. The most often cited reason was the relocation of the family (16 of 38, 42%); other reasons for withdrawal included severe illness (2) and decision to stop once HAART commenced (8). The mean number of hospital admissions was 1.4 in both groups (*P *= 0.83). Twenty-two of the 41 children who were diagnosed with TB were taking daily prophylaxis (*P *= 0.53). Two of these children were diagnosed with multi-drug resistant TB; both were on daily prophylaxis and had mean adherence below 90%.

### Adherence measures and effects of covariates

#### Adherence measured by caregiver self-report

Reports were available for 190 of the children. Ninety-six (51%) were in the daily group. The overall mean adherence was high (94.67%; 95% CI 91.5-97.8) and similar in both groups (daily group adherence 94.69%; 95%CI 92.4-97 and three times a week group adherence 94.67%; 95% CI 88.6-100.7). Similar proportions of children achieved adherence of 90% or higher in the daily (0.81; 95% CI 0.73-0.89) and in the three times per week group (0.80; 95%CI 0.72-0.88).

#### Agreement between caregiver self-report and pill counts

Caregiver self-reports were available for 316 pill count visits (190 children). The numbers of visits at which children were classified as having adhered (achieving adherence ≥ 90%) by pill counts compared to self-reports is shown in Table 2. A statistically significant chi squared was obtained for 2 × 2 comparison of adherence classifications based on the two measures (*P *= 0.002, Fisher's exact test). We calculated kappa statistics, comparing the adherence classification (adherent, ≥ 90%; non-adherent, < 90%) between the two measures per child per visit, as well as the overall adherence classification (mean adherence per child over total study time; classified as adherent, ≥ 90% or non-adherent, < 90%) The two measures had an observed agreement of 76%, kappa = 0.18 for individual measurements (per child per visit); a comparison of the overall adherence gave a slightly lower kappa statistic of 0.1. A summary of the adherence classification based on all available measures is shown in Table 3.

**Table 2:** Agreement between pill counts and caregiver self-report classification of adherent (adherence ≥ 90%) or non-adherent (adherence < 90%).

	**Adherent by pill count**	**Non-adherent by pill count**	**Total**
Adherent by self-report	225 (71%)	56(18%)	281

Non-adherent by self-report	19(6%)	16(5%)	35

Total	244	72	316

Numbers are expressed as percentages of total number of visits (*n *= 316); adherence estimates are based on a single visit evaluation and limited to visits where both measures were used.

* Chi squared 11.76, Fisher's exact test, *p *= 0.002

**Table 3:** Numbers of children classified as adherent (adherence ≥ 90%) or non-adherent (adherence < 90%) by adherence measure used.

**Adherence measure**	**Adherent (%)**	**Non-adherent (%)**	**Total**
Pill counts	217(78.6%)	59(21.4%)	276

Caregiver self-report	153(80.5%)	37(19.5%)	190

Percentages calculated by adherence measure; adherence estimates based on mean adherence per child over follow-up time.

#### Adherence measured by pill counts

**Figure 2:**
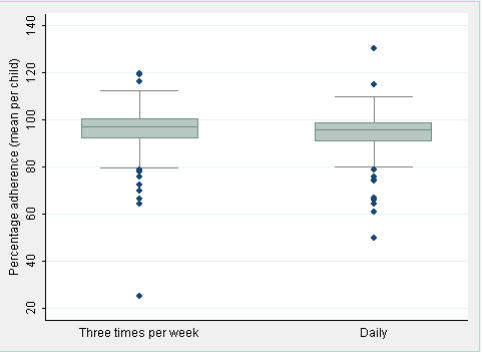
**Average percentage adherence in HIV-infected children comparing three times a week isoniazid to daily treatment calculated from announced pill counts**.

Children attended pill count visits an average of 18 times. Similar numbers of pill count visits were recorded for children receiving daily (mean = 18.7 visits; 95% CI 16.8-20.3) and three times a week prophylaxis (mean = 18.5 visits; 95% CI 17.0-19.9). Overall mean adherence for the study cohort was 94.7% (95% CI 93.5-95.9); 78.6% (217 children) achieved a mean adherence of greater or equal to 90%. A mean adherence of 93.8% was achieved by the daily prophylaxis group (95% CI 92.1-95.6). The three times a week prophylaxis group obtained a similar mean adherence of 95.5% (95% CI 93.8-97.2) The proportion of children considered to be adherent was also similar between the groups: 78% of those receiving daily prophylaxis had a mean adherence measurement of at or above 90% (95% CI 71-85) compared to 79% of those in the three times a week group (95% CI 72-86). Figure 2 compares the average percentage adherence between the two prophylaxis groups.

**Figure 3:**
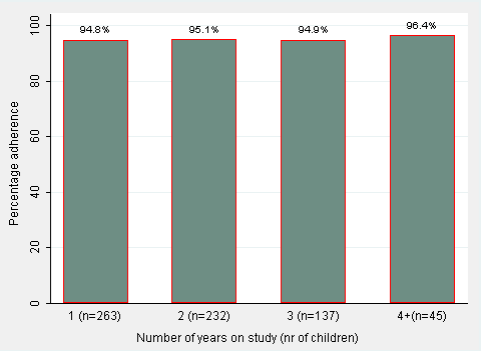
**Mean adherence to isoniazid prophylaxis per year of follow-up time**.

Mean adherence measures per year of follow-up and the number of children contributing to each estimate are shown in Figure 3.

In univariate logistic regression, the prophylaxis group was not associated with adherence greater than or equal to 90% (OR 0.88; 95% CI 0.66-1.17; *P *= 0.38). Age at study visit was significantly associated with adherence: compared to children of less than 1 year of age, 1-4 year olds were less likely to be adherent (OR 0.77; 95% CI 0.6-0.98; *P *= 0.03) and children over 4 years were more likely to be adherent (OR 1.4; 95% CI 1.09-1.8; *P *= 0.008). Household size and the duration of time that a child had been in the study were associated with adherence at the α = 0.1 level. Children who had been in follow-up for more than 1 year were 20% less likely to be adherent than those who had been more recently enrolled (OR 0.82; 95% CI 0.67-1.01; *P *= 0.06; Table 4). In multivariate regression, only age at visit and household size were significantly associated with adherence at the α = 0.05 level after adjusting for prophylaxis group, site, sex, time on study, concurrent use of HAART and disease staging (both CDC immune and clinical staging). Children older than 4 years were almost twice as likely to be adherent as children aged less than 1 year (adjusted OR 1.96; 95% CI 1.16-3.32; *P *= 0.01). In the multivariate regression model, children aged 1-4 years were not significantly more or less likely to be adherent than children aged less than 1 year (*P *= 0.11). Adherence was significantly worse in those from larger households (adjusted OR 0.71; 95% CI 0.54-0.95; *P *= 0.02). After adjusting for other characteristics, children who had been in follow-up for longer than a year were still less likely to be adherent compared to those with less follow-up time, but this failed to reach statistical significance (adjusted OR 0.83; 95% CI 0.66-1.05; *P *= 0.12) No significant interactions were found between prophylaxis group and other covariates.

**Table 4:** Odds ratios for baseline and time-varying predictors of adherence ≥ 90%.

**Baseline characteristics**		**Univariate model** OR (95% CI) P*	**Multivariate model†** AOR (95% CI) P*
Dosing schedule (prophylaxis group)	Three times a week	1	1
	Daily	0.88 (0.66, 1.17) 0.38	0.85 (0.64, 1.11) 0.23

Study site	RCCH	1	1
	TCH	1.25 (0.95, 1.67) 0.11	1.12 (0.82, 1.54) 0.46

Gender	Male	1	1
	Female	1.17 (0.87, 1.57) 0.30	1.11 (0.82, 1.50) 0.51

Number of individuals in house	≤ 5	1	1
	> 5	0.77 (0.57 1.02) 0.07	0.71 (0.54, 0.95) 0.02

Brick house	No	1	-
	Yes	0.98 (0.73, 1.30) 0.87	

Tap water in house	No	1	-
	Yes	1.07 (0.80, 1.43) 0.64	

Flush toilet in house	No	1	-
	Yes	1.08 (0.82, 1.44) 0.57	

Electricity in house	No	1	-
	Yes	0.93 (0.61, 1.41) 0.74	

**Time-varying characteristics**			

Age at visit	< 1 year	1	1
	1-4 years	0.77 (0.6, 0.98) 0.03	1.47 (0.91, 2.37) 0.11
	> 4 years	1.40 (1.09, 1.80) 0.008	1.96 (1.16, 3.32) 0.012

Time on study at visit	< 1 year	1	1
	≥ 1 year	0.82 (0.67, 1.01) 0.06	0.83 (0.66, 1.05) 0.12

CDC clinical	N or A	1	1
stage at visit	B	1.11 (0.85, 1.46) 0.44	0.85 (0.54, 1.34) 0.48
	C	0.86 (0.65, 1.14) 0.30	0.71 (0.43, 1.16) 0.17

CDC immune	1	1	1
Stage at visit	2	0.94 (0.73, 1.20) 0.62	0.96 (0.69, 1.34) 0.83
	3	1.02 (0.79, 1.32) 0.86	1.00 (0.70, 1.44) 0.99

Study drug at visit	Placebo	1	-
	Isoniazid	1.02 (0.73, 1.43) 0.91	-

On HAART at visit	No	1	1
	Yes	1.14 (0.89, 1.45) 0.30	1.08 (0.82, 1.43) 0.56

† Adjusted for group, site, sex, number of people in house, age at visit, concurrent HAART, CDC immune stage at visit, CDC clinical stage at visit, time on study at visit. Two patients excluded from multivariate model due to missing information on number of people in the house and CDC immune staging at follow up.

OR = odds ratio; AOR = adjusted OR; CI = confidence interval; RCCH = Red Cross War Memorial Children's Hospital; TCH = Tygerberg Children's Hospital; CDC = Centers for Disease Control and Prevention; HAART = highly active antiretroviral therapy; * *P *values from Wald tests

## Discussion

Daily INH prophylaxis showed no adherence benefit over the three times per week regimen in our study. As reported previously, the efficacy of the two dosing schedules was equivalent, with both groups experiencing a marked reduction in mortality and TB incidence when on INH as compared to placebo [8]. Furthermore, no toxicity difference was found between the two groups (unpublished data). In our cohort, three times a week INH prophylaxis was therefore not only highly efficacious in reducing mortality and TB in HIV-infected children, but also as easily adhered to and tolerated as daily INH. The use of isoniazid as TB prophylaxis in HIV-infected adults, after the exclusion of active TB, is recommended as part of the World Health Organization's Stop TB strategy [23]. INH prophylaxis is also recommended for children younger than 5 years with proven or suspected latent TB, irrespective of HIV status, although experts advise that HIV-infected children of all ages would benefit [24,25]. Yet the implementation of these measures has been slow, limited by concerns regarding the emergence of TB resistance as well as by economic and health system constraints [1]. For HIV-infected children in whom active TB has been excluded, an intermittent dosing schedule of INH prophylaxis would be cheaper and potentially easier to administer than daily INH. This could, in part, help to address the latter concerns.

Overall adherence to INH among our cohort of children was excellent, with the majority achieving a mean adherence of ≥ 90%. This is in keeping with population HAART adherence rates found in HIV-infected adults and children from similar resource-limited settings [26-28]. There is no gold standard for adherence [29]. As with adherence to HAART, comparing estimates of adherence to TB prophylaxis between studies is complicated by varying definitions of adherence and the use of different adherence tools [11,12]. Traditionally, completion rates (based on taking > 80% of the prescribed doses) have been used to describe adherence to TB prophylaxis [16]. In our study, 88% of children prescribed INH completed a minimum of 6 months of prophylaxis. Only 47.1% of South African HIV-infected adults completed 6 months INH prophylaxis in an operational setting [30], compared to 76% of adults in an Ugandan study [31]. In seminal pre-HIV studies of INH prophylaxis, approximately 70% of Alaskan children were considered to be adherent to study medication based on completion rates [32]. In one prospective and one retrospective study evaluating adherence to INH prophylaxis in operational settings in Cape Town, South Africa, only 15% and 27% respectively of the children completed 5-6 months of INH [33,34]. The majority of these children had an unknown HIV status or were HIV negative. Medication completion rates allow for easy documentation of poor adherence to short-term therapy and are important for programmatic evaluation. However, its use in clinical settings is limited as there is no scope for timely adherence interventions. Our cohort of children and caregivers were provided with substantial adherence support. In particular, the adherence measures we used allowed for immediate feedback and counselling. This allowed the care providers to focus attention on those children and caregivers who were struggling and may, at least in part, explain our high completion rates.

Pill counts may overestimate adherence [17,19]. Other studies of INH have reported pill count-based adherence estimates similar to ours. An Australian study of INH prophylaxis in adolescents recorded a pill count-based adherence estimate of 91% compared to 79% by urine testing, 83% by clinic attendance and 66% by medication event monitoring system [35]. A mean adherence of 85% was reported in a placebo-controlled trial of INH prophylaxis for HIV-infected adults in a setting similar to ours, based on pill counts. These adherence measures correlated well with the reports of patient nominated supervisors [36]. Overestimation of adherence by pill counts is based on falsely assuming that unreturned pills were ingested by the patient. Pill dumping, where containers are returned empty without any medication being taken, is an extreme example, but patients may also discard only a few of the remaining tablets prior to returning the container. In young children, medication doses often have to be repeated by the caregivers due to vomiting, spitting out of medicine or spillage. When more medication is provided than needs to be taken, this can potentially lead to adherence measures of > 100%, even if pill dumping did not occur [37]. In a Ugandan study of paediatric and adult adherence to HAART, unannounced home-based pill counts provided more conservative estimates of adherence than in-clinic announced pill counts (72% of home-based counts achieved adherence > 95% compared to 94% of in-clinic counts) [18]. As our adherence estimates were based on announced pill counts and include some measures above 100%, there was a possible overestimation of adherence. However, the comparison between the prophylaxis groups remains valid. Furthermore, there was a high correlation of pill counts and caregiver self-report, indicating good adherence by multiple measures. A feasibility study of INH prophylaxis in HIV-infected Ugandan adults similarly found high adherence rates by both pill counts (82%) and self-report (85%). In the same cohort, high adherence estimates were also reported by clinic attendance (81%) and urine testing (79%) [31].

In a recent systematic review of paediatric HAART adherence in low- and middle-income countries, self- or caregiver (proxy)-reports were the most frequently used measures of adherence, providing high estimates of adherence (79.5-100%) [11]. Although recall and social desirability bias can result in inflated estimates of adherence, self-report has shown moderate correlation with virological outcomes of patients on HAART [19]. Self-report adherence measures were also valid and reliable in a study on treatment of latent TB among North-American Latino adolescents [38]. Self-report measures are easy to obtain, allow for discussion of obstacles to adherence, are inexpensive and not as labour intensive as some other adherence measures. In our study, unlike most clinical settings, dedicated study pharmacists were responsible for assessing adherence by pill count, whilst study doctors/nurses were responsible for assessing self-report. Therefore, contrary to what would be expected in an operational setting, our clinical staff preferred pill counts to self-reports. Furthermore, caregiver self-reports were often difficult to obtain as the children arrived for study visits with a variety of caregivers, many of whom could not provide information on how medication had been taken in the previous week. Another practical obstacle to measuring adherence encountered by our staff, which may be unique to our setting, was the loss of medicine and diaries in shack fires (fires in informal settlements, usually originating in dwellings where paraffin stoves or open fires are the only sources of heat and energy).

There are three major limitations to our study. First, our main analysis is based on an adherence measure which was only initiated after some children had already been lost to follow up. Attrition is not unexpected in clinical trials and yet it represents an extreme form of non-adherence. Thus, it is possible that we overestimated the overall adherence, as initial defaulters were not accounted for in the analysis. However, as attrition rates were similar in the two prophylaxis groups, this should not influence the comparison between daily and intermittent dosing. Secondly, our estimates are based on measures known to overestimate adherence. However, the measures had a relatively high degree of concordance and our findings are in keeping with the reports on adherence to HAART in HIV-infected children in similar settings. Lastly, we report adherence as measured in a clinical trial setting. Adherence in clinical trial settings is necessarily higher than in operational settings. As clinical trials require informed consent and often preliminary visits before study enrolment, individuals most likely to be non-adherent may well choose to not participate in the trial. Hence, adherence among trial participants is not necessarily predictive of the likely adherence to a similar intervention in the general population. Furthermore, overburdened and unsupported clinic staff members are less likely, and in many cases simply unable, to provide vigilant adherence support such as written or telephonic reminders, meals and payment of transport costs. Such support, however, is integral to a prospective study protocol. Patients are also more likely to be subjected to long waiting times, interrupted drug supplies and worse interpersonal experiences with care providers in resource poor health care centres outside of a study setting [39]. Yet many of these adherence barriers are being addressed and successfully overcome in the context of HIV and HAART [1,26]. Our study suggests that, with adequate adherence support, high levels of adherence to INH prophylaxis can be achieved among selected HIV-infected children despite poor socio-economic circumstances and a prolonged period of prophylaxis.

Medication adherence among HIV-infected children has been extensively studied in the context of HAART. Variable predictors of adherence have been reported in the literature, some seemingly inconsistent [40]. Although our study was not primarily designed to evaluate adherence predictors, we found two associations that might serve to assist health-care workers in identifying children at higher risk for non-adherence. Although adherence in young children depends on the care-giver, age has often been cited as an adherence predictor, with some studies finding younger children to be more adherent and others reporting better adherence in older children [15,41-43]. Many of these studies evaluate age as a continuous measure, without differentiating between the different stages of childhood development. In particular, most studies include adolescents, who frequently struggle with adherence in ways that are different to younger children [40]. Our study included mostly young children, with very few adolescents. Differentiating between infants, toddlers and pre-school children (> 4 years), we found a significantly better adherence among the pre-school children. This may partly be due to the fact that our study drug (INH/placebo) was administered in tablet form. Toddlers, and especially infants, might easily reject not only the taste but also the texture of crushed tablets. Furthermore, it is perhaps easier to negotiate medication adherence in a verbal child than in an infant or toddler. Unfortunately, child-friendly medication in palatable syrup form remains unavailable in many resource-limited settings. The provision of guidance to parents on sources of palatable, safe substances to disguise texture and flavour of life-saving medication should be particularly focused on those with infants and toddlers.

In contrast to a recent report on adherence to HAART among children from a similar South African setting, we did not find any association between adherence and socio-economic factors such as access to water, electricity or a flush toilet [28]. We did, however, find a strong association with our indicator for crowding (that is, the number of people per house). A South African study evaluating intermittent versus daily chemotherapy for the treatment of childhood TB similarly found a significant association between household crowding and adherence, with children from crowded homes achieving a poorer adherence [15]. As children depend largely on caregivers for the administration of treatment, caregiver characteristics are important determinants of adherence [40,42]. Studies from North America have described improved adherence where primary caregivers were not the biological parents, possibly relating to parents' emotions regarding their own HIV status [40,44]. However, caregiver characteristics and their impact on adherence are complex and it is not possible to generalise between different cultural settings. In contrast to reports from industrialized settings, adherence was better among Togolese children whose primary caregivers were their biological parents [45]. A study from South Africa noted misunderstandings between multiple caregivers to be a commonly cited reason for non-adherence to HAART [27]. The association we found between crowding and non-adherence might relate to unmeasured caregiver characteristics, such as having multiple caregivers, rather than to general socio-economic circumstances. Certainly, the complex role of caregiver characteristics in HIV-related paediatric adherence must be explored and refined in the African context. Household size is easily determined and could be a useful tool in assisting health care workers in similar settings to identify children who may be at a higher risk for non-adherence.

## Conclusion

To achieve the Millennium Development Goal 6, target 8 (that is, to halt and reverse TB incidence by 2015), HAART and TB prophylaxis must be affordable and accessible to HIV-infected adults and children in resource-limited settings, including sub-Saharan Africa [23]. We report excellent adherence to three times per week INH and conclude that intermittent dosing could be considered as a more affordable measure for the provision of TB prevention to HIV-infected children.

## Abbreviations

TB: tuberculosis; HAART: highly active antiretroviral therapy; TMP-SMX: trimethoprim-sulphamethoxazole; DSMB: data and safety monitoring board; GEE: generalized estimating equations; CDC: Centers for Disease Control and Prevention.

## Competing interests

The authors declare that they have no competing interests.

## Authors' contributions

SLR was the trial physician for the study, participated in data collection, was responsible for statistical analysis and drafted the manuscript. MFC and HJZ conceived the study, wrote the protocol and grant application and supervised the study. JG supervised the statistical analysis. DLR participated in the statistical analysis and the literature review. LW was responsible for the management of the database. All authors read, approved and contributed to the final manuscript.

## Pre-publication history

The pre-publication history for this paper can be accessed here:



## Supplementary Material

Additional file 1 **Patient adherence diary.** Click here for file

Additional file 2 **Power calculation for comparison of means.** Click here for file
