# Supplementary material for: Adherence to isoniazid prophylaxis among HIV-infected children: a randomized controlled trial comparing two dosing schedules
Source: BMC Med. 2009 Nov 3;7:67. doi: 10.1186/1741-7015-7-67 (PMC2777189; doi:10.1186/1741-7015-7-67)
Supplement: Additional file 1 — Patient adherence diary. [file 1741-7015-7-67-S1.doc]

## Patient Diary

### INH / Cotrimoxazole Study

#### Patient Name: ___________________

#### RXH Folder #: ___________________

Randomisation #: ________________

Date of birth: _____/_____/_____

Date Enrolled: _____/_____/_____

Date Issued: _____/_____/_____

Date Returned: _____/_____/_____

**Please return ALL MEDICINE bottles.**

**Contact Details:**

| **Pharmacy Checklist** | | | | | **Pharmacy Checklist** | | | | |
| --- | --- | --- | --- | --- | --- | --- | --- | --- | --- |
| **Day** | **Date** | **Meds Given** | | **Time Given** | **Day** | **Date** | **Meds Given** | | **Time Given** |
| **26** |  | YES | NO |  | **1** |  | YES | NO |  |
| **27** |  | YES | NO |  | **2** |  | YES | NO |  |
| **28** |  | YES | NO |  | **3** |  | YES | NO |  |
| **29** |  | YES | NO |  | **4** |  | YES | NO |  |
| **30** |  | YES | NO |  | **5** |  | YES | NO |  |
| **31** |  | YES | NO |  | **6** |  | YES | NO |  |
| **32** |  | YES | NO |  | **7** |  | YES | NO |  |
| **33** |  | YES | NO |  | **8** |  | YES | NO |  |
| **34** |  | YES | NO |  | **9** |  | YES | NO |  |
| **35** |  | YES | NO |  | **10** |  | YES | NO |  |
| **Patient Notes** | | | | | **11** |  | YES | NO |  |
|  | | | | | **12** |  | YES | NO |  |
|  | | | | | **13** |  | YES | NO |  |
|  | | | | | **14** |  | YES | NO |  |
|  | | | | | **15** |  | YES | NO |  |
|  | | | | | **16** |  | YES | NO |  |
|  | | | | | **17** |  | YES | NO |  |
|  | | | | | **18** |  | YES | NO |  |
|  | | | | | **19** |  | YES | NO |  |
|  | | | | | **20** |  | YES | NO |  |
|  | | | | | **21** |  | YES | NO |  |
|  | | | | | **22** |  | YES | NO |  |
|  | | | | | **23** |  | YES | NO |  |
|  | | | | | **24** |  | YES | NO |  |
|  | | | | | **25** |  | YES | NO |  |
