# Supplementary material for: Adherence to isoniazid prophylaxis among HIV-infected children: a randomized controlled trial comparing two dosing schedules
Source: BMC Med. 2009 Nov 3;7:67. doi: 10.1186/1741-7015-7-67 (PMC2777189; doi:10.1186/1741-7015-7-67)
Supplement: Additional file 2 — Power calculation for comparison of means. [file 1741-7015-7-67-S2.doc]

**Additional file 1: Power calculation**

**Two sample comparison: mean percentage adherence to isoniazid comparing daily to three times weekly dosing schedule, given sample size n=276 (daily group n=129, three times weekly group n=148); α=0.05 (two-sided test)**

| **Lower of the Two Mean Adherence Percentages** | **Power ( 1-β) for various differences in means (Δ)** | | |
| --- | --- | --- | --- |
|  | **Δ = 5%** | **Δ = 10%** | **Δ = 20%** |
| 60% | 0.98 | 1.00 | 1.00 |
| 70% | 0.98 | 1.00 | 1.00 |
| 80% | 0.98 | 1.00 | 1.00 |
| 90% | 0.98 | 1.00 | - |
